# Comparative Effects of Non-Composted and Composted Sewage Sludge from Wastewater Treatment Plants on the Physiological and Antioxidative Responses of Maize

**DOI:** 10.3390/plants14131955

**Published:** 2025-06-26

**Authors:** Dávid Kaczur, Makoena Joyce Moloi, Seyed Mohammad Nasir Mousavi, Brigitta Tóth

**Affiliations:** 1 Institute of Food Science, Faculty of Agricultural and Food Sciences and Environmental Management, University of Debrecen, 138 Böszörményi St., 4032 Debrecen, Hungary; kaczur.david@unideb.hu; 2 Department of Plant Science, University of the Free State, 205 Nelson Mandela Drive, Park West, Bloemfontein 9301, South Africa; moloimj@ufs.ac.za; 3 Department of Animal Science and Aquaculture, Dalhousie University, Truro, NS B2N 5E3, Canada; nasirmousavi@dal.ca; 4 Institute of Engineering and Agricultural Sciences, University of Nyíregyháza, Sóstói Str. 31/b, 4400 Nyíregyháza, Hungary

**Keywords:** ascorbate peroxidase, chlorophyll, guaiacol peroxidase, proline, plant height, PSII, superoxide dismutase

## Abstract

This study evaluated the physiological and antioxidative responses of maize to non-composted (NCSS) and composted sewage sludge (CSS) from Debrecen and Kecskemét, applied at 25%, 50%, and 75% (m/m) concentrations. Measurements were taken 21 and 35 days after sowing (DAS). Debrecen NCSS significantly enhanced plant height at all concentrations and at both sampling times, while higher doses of Kecskemét NCSS reduced growth by 35 DAS. Chlorophyll-a, chlorophyll-b, and carotenoid contents were notably enhanced by Kecskemét treatments, especially at lower concentrations, whereas Debrecen treatments showed less effect. Photosystem II (PSII) efficiency parameters varied with origin: Kecskemét NCSS notably increased minimal fluorescence (F_o_), while Debrecen CSS occasionally reduced maximum fluorescence (F_m_) and variable fluorescence (F_v_) at 75% dose (21 DAS). The superoxide dismutase activity (SOD) was significantly elevated in Kecskemét treatments—by 101%, 148% and 149% at 25%, 50% and 75% CSS applications. Correlation analysis further highlighted that NCSS treatments often showed negative associations between plant height and chlorophyll content but positive correlations with antioxidant activity. In contrast, CSS treatments promoted balanced physiological responses. The results support the importance of sludge origin and application rate and suggest that composted sludge can be a safe, sustainable amendment when managed appropriately.

## 1. Introduction

In 2023, the world generated approximately 2.1 billion tons of municipal solid waste, according to reports from the United Nations, and this amount is projected to rise significantly, reaching 3.8 billion tons by 2050 [1]. A large portion of this waste includes untreated wastewater, which contributes to environmental pollution and health risks. Currently, ∼380 billion m^3^ (380 trillion liters) of wastewater is generated globally every year [2]. Wastewater generation varies by region and sector, with industrial and agricultural activities being major contributors. Globally, a significant portion of wastewater is discharged without proper treatment, leading to water pollution that affects ecosystems and human health. Only 24% of the total wastewater generated from households and industries is treated before its disposal in rivers or reused in agriculture. One of the most important issues linked with wastewater generation is the residual presence of pathogenic microorganisms, which pose potential health hazards to consumers when they enter the food chain. The other threat of using wastewater is its heavy metal content [3].

Such contaminants can be found in sewage sludge, also called biosolids. Sewage sludge is the semi-solid byproduct left over after the treatment of wastewater at sewage treatment plants. The effects of sewage sludge on plants can be both beneficial and harmful, depending on the type, amount, and method of application. It is a valuable fertilizer that can enrich the soil with organic matter and nutrients, improving soil quality and increasing crop yields [4]. It also enhances structure, chemical properties, and microbial activity in the soil [5,6]. Additionally, its organic matter and nutrients may stimulate plant growth and increase metabolic activity [7]. In contrast to its good properties, sewage sludge can contain heavy metals like lead, cadmium, and nickel, which can be absorbed by plants and contaminate the food chain [8]. Additionally, it contains organic pollutants that pose environmental and health risks like per- and polyfluoroalkyl substances (PFAS) and brominated flame retardants [9]. Heavy metals can negatively impact the soil’s biological functions, such as the activity of enzymes and the size and diversity of soil microbes [10].

When plants absorb heavy metals or other pollutants from sewage sludge, they often produce more reactive oxygen species (ROS). In excess, ROS can cause oxidative stress, triggering the plant’s antioxidant defense system [11]. Sewage sludge influences antioxidant enzyme activity in plants due to stress from heavy metals, organic pollutants, or its nutrient-rich composition [12]. Antioxidant enzymes such as superoxide dismutase (SOD), catalase (CAT), and peroxidase (POD) help protect plants by neutralizing ROS under stress conditions [13]. Studies on wheat, maize, and pakchoi cabbage showed that sewage sludge application increases SOD, CAT, and POD activity, suggesting an enhanced antioxidant defense response to heavy metal or aluminum stress [7,14,15]. Sludge with high heavy metal concentrations led to excessive ROS production and increased antioxidative enzyme activity in maize [16]. However, in some cases, severe metal toxicity inhibited enzyme function. Dhanya et al. [17] found that chlorophyll content gradually decreased with increasing sewage sludge concentrations of 50% to 75%, while carotenoid content increased.

To mitigate the negative effects of sewage sludge, treatment of the sludge and appropriate farm management practices can be used. One possible treatment is composting. This process converts organic materials into a nutrient-rich, biologically stable soil amendment or mulch through natural decomposition under a controlled, aerobic (oxygen-required) environment [18]. Sewage sludge compost can significantly influence antioxidant enzyme activity in plants, often enhancing their resilience to environmental stress [15]. By providing essential nutrients and inducing mild stress responses, it promotes the activation of antioxidant enzymes like SOD, CAT, and POD, which are crucial for maintaining cellular homeostasis. However, the presence of contaminants in untreated or poorly managed compost poses risks that can disrupt these beneficial effects [19].

The heavy metal content in composted and non-composted sewage sludge is usually different. One reason is that sewage sludge is dewatered, so metals originally present in the liquid effluent become highly concentrated in the solid fraction. Compost feedstocks are more diverse and mostly consist of plant-derived material with very low heavy metal levels, so even after microbial mineralization, metals remain diluted [20]. In addition, during the composting process, microbial activity promotes humification, which binds metals into stable organic complexes and reduces their mobility and bioavailability [21]. In contrast, non-composted sludge lacks this stabilization stage, so metals remain in more labile forms and at higher total concentrations per unit dry matter [22]. Furthermore, when producing compost, the feedstock is often pre-screened to remove foreign materials and inorganic contaminants [23]. In contrast, sewage sludge is less selective—heavy metals from industrial discharges cannot easily be separated during typical sludge treatment processes [24].

Evaluating plant responses to sewage sludge amendments requires a multifaceted approach, as such amendments influence both physiological and biochemical plant processes. The following parameters are commonly used because they serve as sensitive indicators of plant health, nutrient status, and stress responses. Plant height is a simple yet integrative growth parameter that reflects overall plant vigor and productivity [25]. It responds to both beneficial effects (e.g., increased nutrient availability from organic matter) and detrimental effects (e.g., heavy metal toxicity or nutrient imbalance) of sludge amendments [26].

Photosynthetic pigments are essential for light absorption and energy transfer in photosynthesis [27]. Changes in chlorophyll and carotenoid content can indicate alterations in plant metabolic activity due to nutrient availability or toxicity caused by heavy metals in sludge [28]. Chlorophyll degradation is often associated with stress [29], while increased pigment content reflects enhanced photosynthetic capacity and nutrient availability [30]. Carotenoids also play a protective role by quenching reactive oxygen species (ROS) [31]. Photosystem II efficiency parameters (such as F_v_/F_m_) are reliable indicators of photosynthetic performance and stress impact at the photochemical level [32]. Heavy metals or other pollutants from sewage sludge may impair electron transport, leading to decreased efficiency [23]. F_v_/F_m_ ratio is used to assess the maximum quantum efficiency of PSII and serves as a diagnostic tool for photoinhibition and stress [33,34]. Proline accumulation is a common response to various abiotic stresses, including salinity and heavy metal exposure [35]. It acts as an osmoprotectant, stabilizing proteins and membranes and scavenging free radicals [36]. Elevated proline levels often signal oxidative or osmotic stress resulting from sludge-related toxicity [37]. Sewage sludge can introduce heavy metals and other stressors, which promote ROS production in plants [12]. Antioxidant enzymes such as superoxide dismutase (SOD), catalase (CAT), and peroxidase (POD) are critical for detoxifying ROS and protecting cellular components [38]. Elevated enzyme activity often reflects activation of defense mechanisms against oxidative stress [39]. In summary, these parameters offer a comprehensive view of how sewage sludge amendments affect plant function, enabling researchers to distinguish between nutrient-enriching effects and toxicity-related stress responses

The hypothesis was that the application of increasing concentrations of composted sewage sludge enhances the measured parameters in maize compared to the control, while in the case of non-composted sewage sludge, we assumed the opposite effect. The goal of this study was to examine the effects of non-composted (NCSS) and composted sewage sludge (CSS) from two sources (Debrecen and Kecskemét) at varying concentrations (25%, 50%, 75% m/m) over two sampling times (21 and 35 days after sowing, DAS) on main physiological and antioxidative responses of maize. As the global demand for sustainable farming practices grows, integrating sewage sludge into crop management systems, supported by rigorous research and policy frameworks, holds promise for improving agricultural productivity while addressing waste management challenges.

## 2. Results

The goal of this study was to study the effects of composted and non-composted sewage sludge (CSS and NCSS) on maize’s photosynthetic efficiency, morphological, and antioxidative characteristics. For this, two composted and two non-composted sewage sludges from two Hungarian cities (Debrecen and Kecskemét) were used. For sampling times, 21 and 35 days after sowing (DAS) were chosen because at 21 DAS, maize is typically at the early growth stage, characterized by the development of three to four fully expanded leaves. During this period, plants are highly responsive to external inputs such as fertilizers or organic amendments, making it an ideal point for assessing early physiological and biochemical changes, including pigment accumulation, antioxidant enzyme activities, and oxidative stress markers [40]. By 35 DAS, maize generally reaches the mid-vegetative stage, where rapid leaf expansion and biomass accumulation occur. Physiological markers such as chloroplast pigments, fluorescence, proline accumulation, and antioxidant enzyme activities (SOD, POD, and APX) are especially responsive during these stages, making 21 and 35 DAS optimal for monitoring stress and adaptation responses [41].

### 2.1. Plant Height

**Figure 1:**
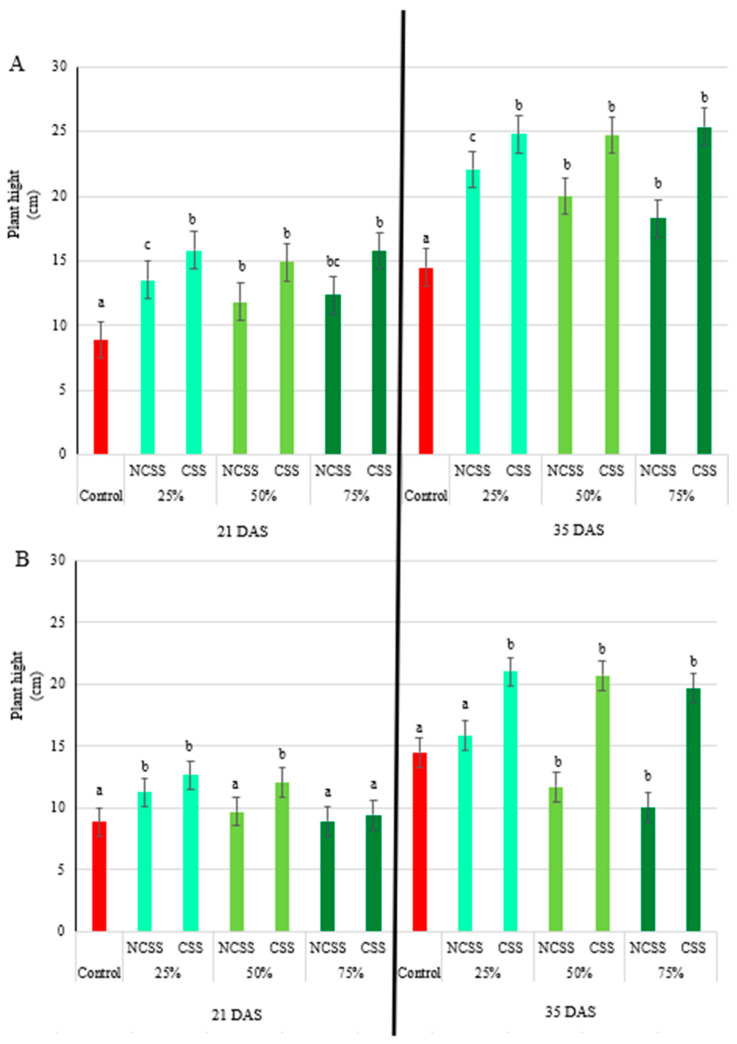
Effects of Debrecen (**A**) and Kecskemét (**B**) composted (CSS) and non-composted sewage sludge (NCSS) on maize plant height (cm) measured at 21 and 35 days after sowing (DAS). Values represent mean (*n* = 5). Figure legends 25, 50, and 75% (m/m) represent the applied concentrations of the NCSS and CSS. Lowercase letters represent significant differences relative to control (*p* ≤ 0.05).

At 21 days after sowing (DAS), plants treated with non-composted sewage sludge (NCSS) from Debrecen were significantly taller than the control across all application rates. The 25% NCSS treatment from Debrecen led to the greatest height increase, yielding a 1.52-fold enhancement compared to the control. In contrast, plants treated with Kecskemét NCSS showed a significant height increase only at the 25% application rate, with a 1.26-fold improvement. By 35 DAS, Debrecen NCSS-treated plants maintained their superior height relative to controls, with 25% and 50% treatments producing 1.50- and 1.39-fold increases. However, higher application rates of Kecskemét NCSS (50% and 75%) negatively impacted growth, reducing plant height to 0.81- and 0.69-fold of the control (Figure 1A).

For composted sewage sludge (CSS), Debrecen treatments at 21 DAS led to significantly increased plant heights at all application levels, with fold increases of 1.78, 1.67, and 1.77 for 20%, 50%, and 75% doses, respectively. Kecskemét CSS, on the other hand, showed effects only at 25% (1.42-fold) and 50% (1.36-fold) application rates. Overall, all plants treated with Debrecen compost showed increased height, while the Kecskemét compost had the most favorable effect at lower application rates. By 35 DAS, Debrecen CSS treatments consistently promoted growth, with all doses resulting in at least a 70% increase in height. Kecskemét CSS also enhanced height, with fold increases of 1.44, 1.42, and 1.36 for the 25%, 50%, and 75% treatments, respectively (Figure 1B).

### 2.2. Photosynthetic Pigments Content

At 21 DAS, pigment analysis revealed no significant change in Debrecen NCSS-treated plants, except for a total chlorophyll increase at the 50% treatment. In contrast, Kecskemét NCSS treatments significantly elevated levels of chlorophyll-a, chlorophyll-b, carotenoids, total chlorophyll, and total chl/car across all concentrations, with the exception of the chlorophyll-a/b ratio. By 35 DAS, Debrecen NCSS continued to influence total chlorophyll positively, showing more than a 2.3-fold increase at all application rates. Kecskemét NCSS treatments induced a significant increase in chlorophyll-a, and chlorophyll-b (at 25% and 50%), carotenoid, and total chlorophyll, with the highest effect at the 25% level (Table 1).

At the first sampling time (21 DAS), the Debrecen CSS treatments led to a significant reduction in chlorophyll-b and total chlorophyll content at the 25% and 50% application rates. Specifically, chlorophyll-b levels decreased by 1.3- and 1.4-fold, while total chlorophyll content declined by 1.4- and 1.3-fold, respectively. In contrast, all Kecskemét CSS-treatments significantly enhanced chlorophyll-a content, showing 1.49-, 1.63-, and 1.53-fold increases for the 25%, 50%, and 75% doses, respectively. Chlorophyll-b content in Kecskemét CSS-treated plants also showed significant increases at the 25% and 50% doses, rising by 1.48- and 1.66-fold, respectively. Carotenoid content was consistently elevated across all Kecskemét CSS treatments, while increases of 2.28-, 2.12-, and 2.14-fold at the 25%, 50%, and 75% doses, respectively.

At the second sampling time (35 DAS), both Debrecen and Kecskemét CSS treatments resulted in significant increases in chlorophyll-a content. At the 25% dose, chlorophyll-a values increased 2.03-fold at both origins (Debrecen and Kecskemét). For the 50% dose, values rose by 1.94-fold (Debrecen) and 1.95-fold (Kecskemét), while the 75% dose resulted in 1.90- (Debrecen) and 2.11-fold (Kecskemét) increases relative to the control. Chlorophyll-b content also significantly increased in all treatments for both CSS origins. In Debrecen CSS-treated plants, the 25%, 50%, and 75% applications led to 1.67-, 1.51-, and 1.51-fold increases compared to the control, respectively. For Kecskemét CSS, chlorophyll-b increases were even more pronounced: 2.32-, 1.89-, and 2.11-fold increase. Carotenoid content at 35 DAS showed significant changes only in specific treatments. A notable increase was observed for the 25% Debrecen CSS dose (1.29-fold). In contrast, all Kecskemét CSS treatments led to significant carotenoid enhancement, with increases of 1.43-, 1.32-, and 1.37-fold at the 25%, 50%, and 75% application rates, respectively (Table 2).

### 2.3. Photosynthetic Efficiency Parameters

No significant differences in photochemical parameters were observed at 21 DAS under Debrecen NCSS treatments. However, Kecskemét NCSS significantly increased basal fluorescence (F_o_) at the 75% dose (1.20-fold). By 35 DAS, Debrecen NCSS showed no changes in photochemistry, whereas Kecskemét NCSS showed significantly increased F_o_ values across all doses, by 1.32-fold (25% and 50%) and 1.46-fold (75%) compared to the control (Table 3).

For CSS, Debrecen 75% treatment at 21 DAS significantly decreased F_o_ (0.85-fold), maximal fluorescence (Fₘ), and variable fluorescence (Fᵥ) parameters (both 0.87-fold). Kecskemét CSS at 25% also led to a slight yet significant reduction in Fₘ (0.96-fold) and Fᵥ (0.97-fold). At 35 DAS, Debrecen CSS at 50% significantly increased F_o_ (1.15-fold), Fₘ and Fᵥ (both 1.16-fold), while Kecskemét CSS treatments had no significant effect on any photochemical parameter (Table 4).

### 2.4. Proline Concentration

**Figure 2:**
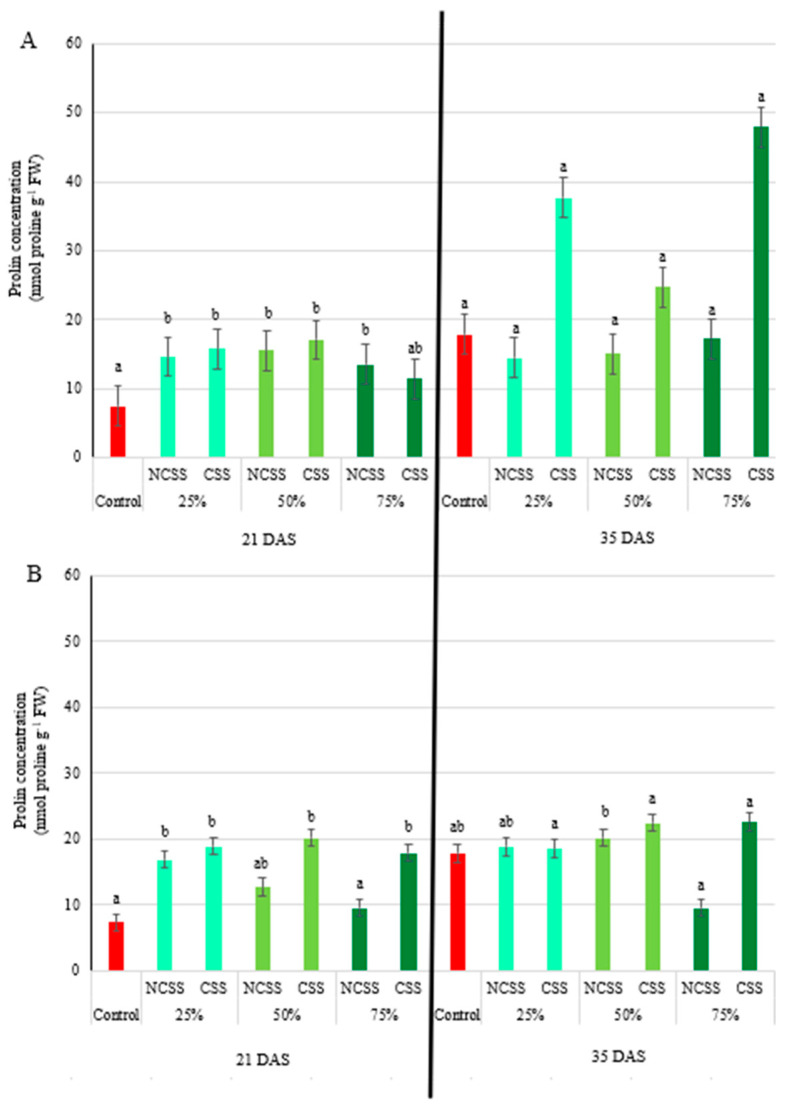
Effects of Debrecen (**A**) and Kecskemét (**B**) composted (CSS) and non-composted sewage sludge (NCSS) on proline concentration (nmol proline g^−1^ FW) measured at 21 and 35 days after sowing (DAS). Values represent mean (*n* = 5). Figure legends 25, 50, and 75% (m/m%) represent the applied concentrations of the NCSS and CSS. Lowercase letters represent significant differences relative to control (*p* ≤ 0.05). FW: fresh weight.

At 21 DAS, Debrecen NCSS significantly increased proline levels at all concentrations: 1.97-, 2.10-, and 1.82-fold for 25%, 50%, and 75% treatments, respectively. Kecskemét NCSS only showed a significant increase at the 25% concentration (2.29-fold). By 35 DAS, proline levels in all NCSS treatments had returned to control levels (Figure 2A). Debrecen CSS induced significant proline accumulation at 21 DAS, particularly at 25% (2.13-fold) and 50% (2.31-fold). Kecskemét CSS resulted in ever greater increases—2.57-, 2.73-, and 2.43-fold for the 25%, 50%, and 75% treatments, respectively. However, by 35 DAS, no significant differences were observed (Figure 2B).

### 2.5. Antioxidative Enzymes Activity

**Figure 3:**
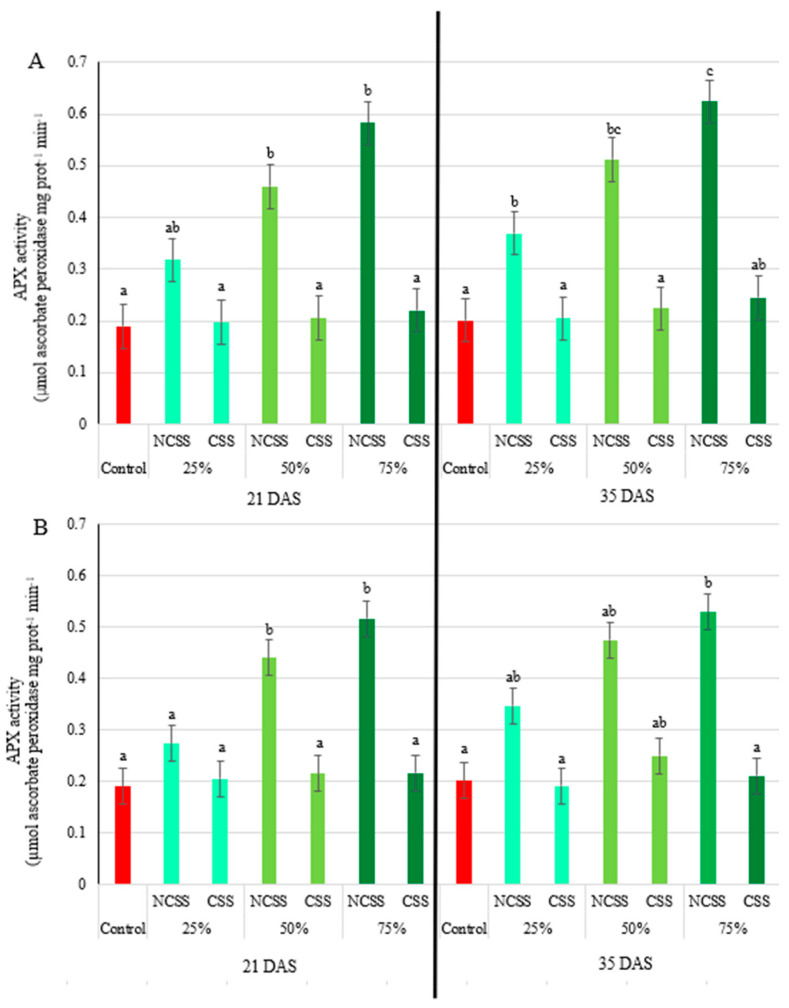
Effects of Debrecen (**A**) and Kecskemét (**B**) composted (CSS) and non-composted sewage sludge (NCSS) on the ascorbate peroxidase (APX) activity (µmol ascorbate peroxidase min^−1^ mg prot^−1^) measured at 21 and 35 days after sowing (DAS). Values represent mean (*n* = 5). Figure legends 25, 50, and 75% (m/m%) represent the applied concentrations of the NCSS and CSS. Lowercase letters represent significant differences relative to control (*p* ≤ 0.05). FW: fresh weight.

At 21 DAS, ascorbate peroxidase (APX) activity was significantly higher in response to the 50% and 75% NCSS from both Debrecen and Kecskemét. In both cases, the 75% dose induced the most pronounced increase in activity: 3.07-fold higher than the control for Debrecen NCSS, and 2.72-fold higher for Kecskemét NCSS. The 25% treatment, however, did not produce a statistically significant change in APX activity, regardless of sludge origin. By 35 DAS, all NCSS treatments—across both locations—resulted in significantly increased APX activity compared to the control. Notably, the 75% Debrecen NCSS treatment not only showed the highest activity (3.09-fold above control) but also differed significantly from the 25% and 50% doses, underscoring a dose-dependent effect. Even the smallest increase at this stage exceeds 70% relative to the control Figure 3A. In contrast, no significant change in APX activity was observed in response to any CSS treatment—whether from Debrecen or Kecskemét—at either sampling time (Figure 3B).

At 21 DAS, all Debrecen NCSS concentrations significantly enhanced peroxidase (POD) activity, with 2.51-, 3.44-, and 5.02-fold for the 25%, 50% and 75% concentrations, respectively. The 75% treatment also significantly differed from the lower concentrations. Kecskemét NCSS showed increased POD activity only at the 50% (1.78-fold) and 75% (2.75-fold) doses.

**Figure 4:**
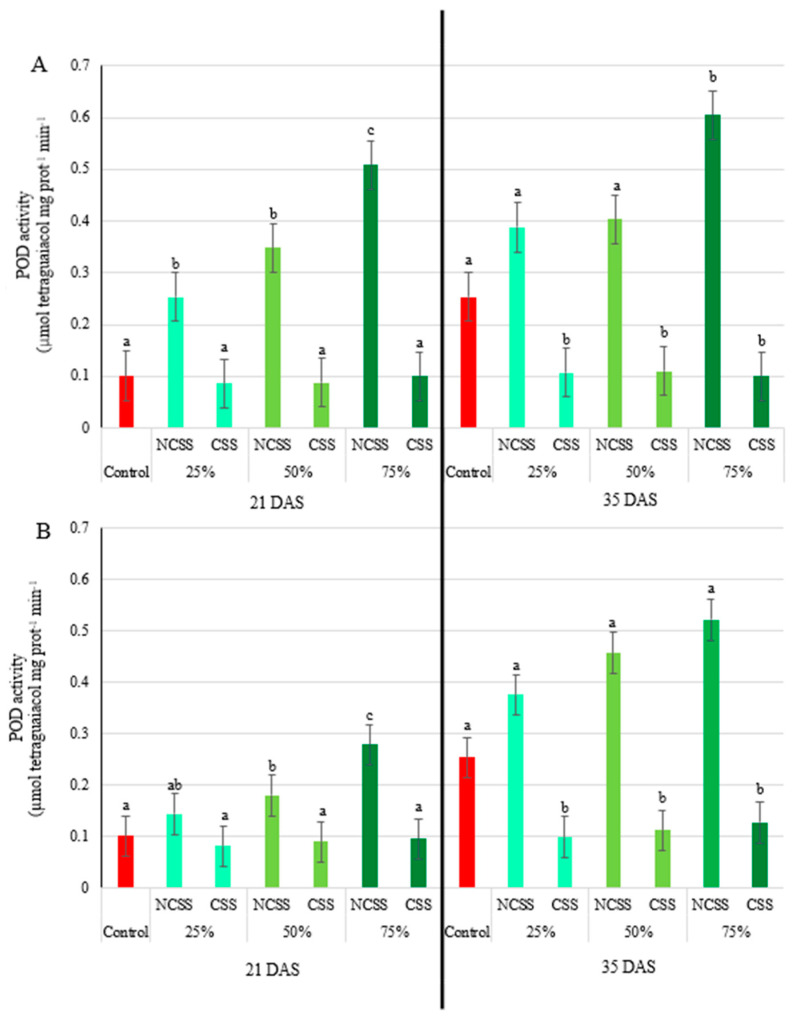
Effects of Debrecen (**A**) and Kecskemét (**B**) composted (CSS) and non-composted sewage sludge (NCSS) on the peroxidase (POD) activity (µmol tetraguaiacol mg prot^−1^ min^−1^) measured at 21 and 35 days after sowing (DAS). Values represent mean (*n* = 5). Figure legends 25, 50, and 75% (m/m%) represent the applied concentrations of the NCSS and CSS. Lowercase letters represent significant differences relative to control (*p* ≤ 0.05). FW: fresh weight.

At 35 DAS, only the 75% Debrecen NCSS treatment maintained significantly elevated POD activity (2.38-fold). No significant changes were observed in Kecskemét NCSS treatments at this stage (Figure 4A). In contrast, no significant changes in POD activity were detected at 21 DAS in CSS-treated plants. However, by 35 DAS, all CSS treatments from both locations significantly decreased POD activity. Debrecen CSS reduced activity by approximately 40% at all concentrations, while Kecskemét CSS led to 0.32-, 0.35-, and 0.37-fold reductions at the 25%, 50%, and 75% application rates, respectively (Figure 4B).

**Figure 5:**
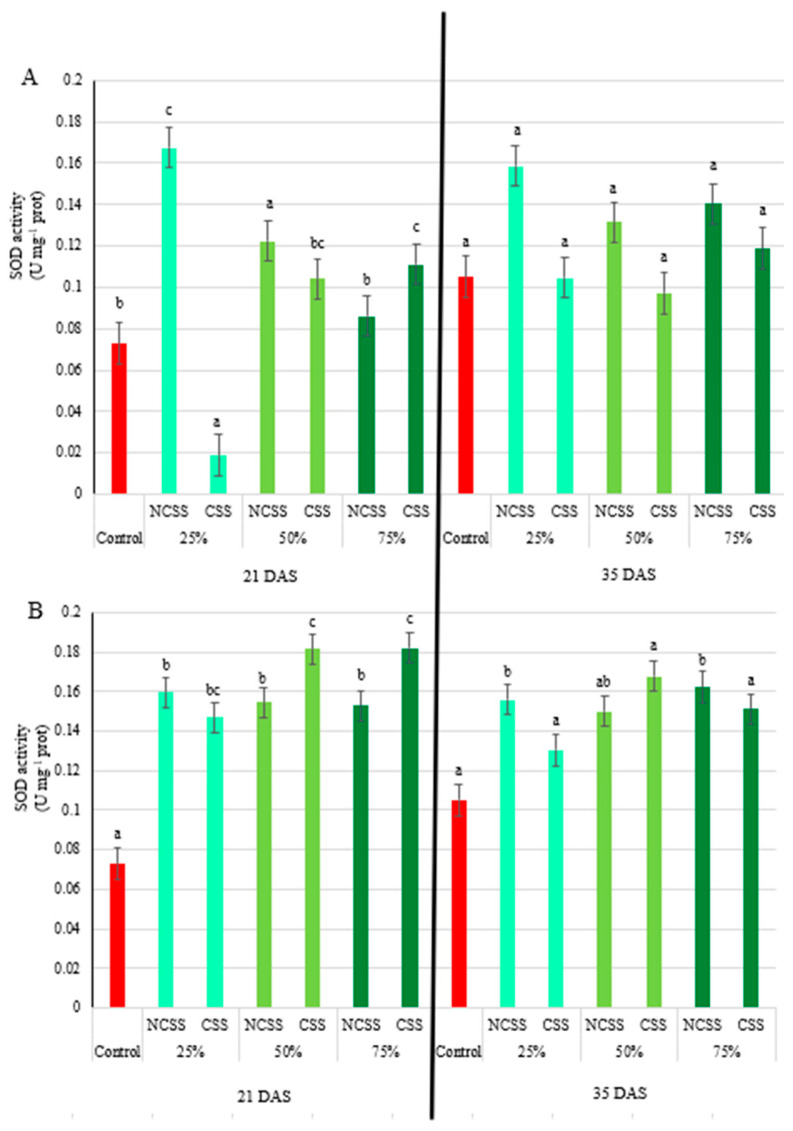
Effects of Debrecen (**A**) and Kecskemét (**B**) composted (CSS) and non-composted sewage sludge (NCSS) on the superoxide dismutase (SOD) activity (U mg^−1^ protein) measured at 21 and 35 days after sowing (DAS). Values represent mean (*n* = 5). Figure legends 25, 50, and 75% (m/m%) represent the applied concentrations of the NCSS and CSS. Lowercase letters represent significant differences relative to control (*p* ≤ 0.05).

At 21 DAS, Debrecen NCSS significantly increased SOD activity at 25% and 50% application rates. In contrast, Kecskemét NCSS significantly elevated SOD activity at all concentrations. By 35 DAS, no significant differences were observed for Debrecen NCSS, while Kecskemét NCSS continued to increase SOD activity—1.48-fold at 25% and by 1.54-fold at 75% (Figure 5A).

Debrecen CSS at 21 DAS induced contrasting responses: a 25% reduction in SOD activity at the 25% dose and a 52% increased at the 75% dose. Kecskemét CSS consistently elevated SOD activity at all concentrations—2.02-, 2.49-, and 2.50-fold, respectively (Figure 5B).

## 3. Discussion

In this study, the effects of composted (CSS) and non-composted sewage sludge (NCSS) on the physiological and biochemical characteristics of maize were investigated, with a particular focus on plant growth, photosynthetic efficiency, and antioxidant enzyme activity. The findings highlight the different responses of maize to treatments with sludge from two origins (Debrecen and Kecskemét) at varying concentrations (0%, 25%, 50%, and 75%) and sampling time (21 and 35 days after sowing, DAS).

The positive effects of Debrecen NCSS on maize height at the early vegetative stage (21 DAS) and continued growth at 35 DAS (Figure 1) align with findings from previous studies that report beneficial impacts of sewage sludge on plant growth [42,43,44,45,46]. Specifically, a study by Singh and Agrawal [47] found that non-composted sewage sludge promotes growth in mung beans. Compared to plants treated with chemical fertilizer alone, maize and faba bean supplemented with sewage sludge compost performed considerably better for growth [48]. The 50% increase in height observed under 25% concentration of Debrecen NCSS treatment is consistent with findings by Alvarenga et al. [49], where moderate application of sewage sludge significantly enhanced maize height, potentially due to the availability of macro and micronutrients. In contrast, the negative effects of higher concentrations of Kecskemét NCSS (50% and 75%) on plant growth observed at 35 DAS were similar to those found by Natal-da-Luz [50], who reported that high doses of non-composted sewage sludge might cause toxicity, leading to inhibited growth due to salt accumulation or the presence of heavy metals. This effect was particularly evident under the 75% concentration, where a significant decrease in plant height was measured. Koutrousbas et al. [46] observed that the plant height responded linearly to sewage sludge application rate, while in this study was not significantly decreased the plant height with the linear relationship of the applied NCSS and CSS concentrations at all treatments. The increase in chlorophyll-a, chlorophyll-b, and carotenoid contents in maize treated with Kecskemét NCSS at 21 DAS is consistent with Hao et al. [7], who demonstrated that organic amendments such as sewage sludge improve the chlorophyll content in rice by enhancing nutrient availability and reducing oxidative stress. The ratio of leaf carotenoid to chlorophyll is an indicator of photosynthesis, development, and stress response [51]. Under normal growing conditions, both Car and Chl are low at the beginning of the growing season and high at the peak of the growing season. This means that under these conditions, the ratio of the two is static, and a fixed value can be assumed. However, under stress conditions, Car tends to decline more slowly than Chl, leading to deviation from such a static fixed value [52,53,54]. The chlorophyll a/b ratio is a good indicator of leaf senescence, stress, and damage to the photosynthetic apparatus [55]. The leaf chlorophyll a/b ratio ranged from 0.85 to 3.82 (Table 1 and Table 2), which is mostly lower than the average value (3.0) under normal growing conditions [56]. Furthermore, the positive significant correlation between chlorophyll-a and carotenoids in this study (r = 0.938 ** Table S1, r = 0.987 ** Table S2, r = 0.556 ** Table S4) reflects the relationship noted by Zhu et al. [57], who reported that higher chlorophyll levels in tomato are often associated with increased carotenoid synthesis, suggesting improved photosynthetic capacity under organic fertilization. However, Dhanya et al. [17] suggested that excessive levels of sewage sludge could lead to chlorosis or a reduction in chlorophyll content, which aligns with the findings of reduced pigment levels in Debrecen NCSS at higher concentrations.

Regarding the photosynthetic efficiency, increased F_o_ values in Kecskemét NCSS treatments observed in this study are indicative of enhanced light absorption, which is consistent with Hao et al. [7], who found that compared to nitrogen fertilizer treatments, F_o_, F_m_, F_v_, and F_v_/F_m_ parameter in sewage sludge-derived nutrients + biostimulants treatment were improved by 51.68%, 52.81%, 53.08%, and 1.70%, respectively. This result shows that sludge-derived nutrients + biostimulants treatment could promote plant photosynthesis by improving the photosystem (enhancing light capture and accelerating electron transport). In addition, the maximum quantum yield of photosystem II (F_v_/F_m_) was reduced in basil leaves grown on eroded soil. However, the addition of sewage sludge to this soil increased F_v_/F_m_ to a value close to normal (0.83) [45].

Proline is a well-known osmoprotectant that accumulates in plants under various abiotic stresses, including nutrient imbalance and oxidative stress [58]. At the first sampling time (21 DAS), all concentrations of the Debrecen NCSS induced significant increases in proline content, with the 50% treatment resulting in the highest increase (110% over the control) (Figure 2). This suggests an early stress response or metabolic stimulation triggered by the sludge amendments. In contrast, the Kecskemét NCSS only induced a significant increase at the 25% level (128% over control), indicating possible variability in the composition or nutrient availability of the two sludge types. At the second sampling time (35 DAS), the lack of significant differences in proline content for both NCSS and CSS treatments suggests that the initial stress or stimulation was either transient or that plants had adapted to the conditions. Similar patterns of early proline accumulation followed by normalization have been reported under organic fertilization scenarios [59].

Ascorbate peroxidase plays a key role in scavenging reactive oxygen species (ROS), particularly hydrogen peroxide, and is thus a crucial enzyme in the plant antioxidative defense system [60]. At 21 DAS, significant increases in APX activity were observed for the 50% and 75% NCSS treatments from both locations, with the Debrecen NCSS 75% treatment reaching a peak of 208% above control levels. This suggests an induced oxidative response and a potential triggering of systemic acquired resistance [61]. However, at 35 DAS, significant differences were observed across all NCSS treatments, with even the lowest concentration leading to over 70% increases in APX activity. The sustained increase in APX activity implies prolonged oxidative stress reduction due to organic amendments. Conversely, no significant changes were detected for CSS treatments at either sampling time, implying a lower impact on oxidative stress pathways or differences in bioavailability of active compounds in composted materials versus non-composted sludge. Peroxidase activity, another indicator of stress tolerance [62], showed substantial increases under NCSS treatments at 21 DAS, especially in the Debrecen samples. By 35 DAS, the increased peroxidase activity persisted only in the Debrecen NCSS at the highest concentration, suggesting a lasting effect under this treatment. The composted treatments (CSS), however, showed no significant differences from the control, supporting the idea that composting may stabilize organic matter and reduce bioactive stress-inducing compounds [41]. Superoxide dismutase (SOD) is a key antioxidant enzyme that catalyzes the dismutation of superoxide radicals into oxygen and hydrogen peroxide, playing a crucial role in the plant’s defense system against oxidative stress [63]. The variations observed in SOD activity in response to different compost and sewage sludge treatments reflect the dynamic nature of oxidative stress and the antioxidant defense system in plants under organic amendment regimes. At 21 days after sowing (DAS), significant increases in SOD activity were detected in response to the Debrecen NCSS at 25% and 50% concentrations, suggesting an early oxidative response potentially induced by elevated nutrient availability or the presence of reactive compounds [60]. These findings align with previous studies indicating that low to moderate doses of sewage sludge can stimulate ROS production and subsequently activate the antioxidant defense system [41]. Interestingly, all concentrations of Kecskemét NCSS led to significantly elevated SOD activity at 21 DAS, with increases ranging from 48% to 54% (for 25 to 75% dosages). This stronger and broader response could be attributed to differences in sludge composition, such as higher levels of organic matter, micronutrients, or pro-oxidant compounds in the Kecskemét material [63]. Such variation highlights the importance of sludge origin and treatment in influencing plant physiological responses. By 35 DAS, SOD activity returned to control levels across all treatments, indicating that the oxidative stress or stimulation induced by the amendments was transient. This decline in enzyme activity suggests that the plants had either adapted to the initial stress or that the availability of reactive compounds had diminished over time due to microbial degradation or plant uptake [64,65]. The response pattern to composted sludge was notably different. At 21 DAS, the Debrecen CSS treatments showed a mixed trend: while the 25% concentration resulted in a 25% decrease in SOD activity relative to the control, the 75% concentration led to a 52% increase. This biphasic response could be due to varying levels of stabilized organic matter and microbial activity influencing ROS signaling and antioxidant demand [66]. In contrast, the Kecskemét CSS induced a consistently strong increase in SOD activity across all concentrations, with enhancements of 101%, 148%, and 149% for the 25%, 50%, and 75% treatments, respectively. This suggests a potent stimulation of the antioxidative defense system, possibly due to bioactive compounds or humic substances formed during composting, which have been shown to enhance enzymatic antioxidant activity in plants [67].

While no significant differences in oxidative enzyme activities were observed at the first sampling, by 35 DAS, a significant reduction in peroxidase activity was recorded for both Debrecen and Kecskemét CSS treatments. The values were 40–50% lower than the control, indicating a potential stress-alleviating or growth-regulating effect of the composted material like CSS [67]. This contrasts sharply with the stimulating effect of NCSS and points toward composts being more suited for long-term soil fertility management rather than stress induction. Furthermore, the absence of significant differences at 35 DAS for both CSS and NCSS treatments again suggests a temporal regulation of SOD activity, with early-stage stimulation giving way to adaptation or reduced stress intensity. The lower peroxidase (POD) activity observed in plants treated with composted sewage sludge compared to non-composted sewage sludge (Figure 4) can be attributed to differences in organic matter stability, heavy metal bioavailability, and phytotoxicity, which directly affect plant oxidative stress levels. Non-composted sewage sludge often contains higher levels of bioavailable heavy metals (e.g., Cd, Pb, Zn, Cu), which can trigger oxidative stress by generating excessive reactive oxygen species (ROS) in plant tissues. In response, plants activate antioxidant enzymes like peroxidase to detoxify hydrogen peroxide (H_2_O_2_) and limit oxidative damage. Composting stabilizes heavy metals by forming insoluble complexes with organic matter, thus reducing their mobility and uptake by plants [49]. As a result, the ROS burden in plants decreases, leading to lower peroxidase activity. In addition, an inconsistent effect on SOD activity was observed in Figure 5B, where the activity was 8.48-fold higher in plants treated with 25% Kecskemét CSS compared to those treated with 25% Debrecen CSS at 21 DAS. This may be attributed to the fact that superoxide dismutase (SOD) is one of the most sensitive antioxidant enzymes in plants, serving as the first line of defense by catalyzing the dismutation of superoxide radicals (O_2_•^−^) into the less reactive hydrogen peroxide (H_2_O_2_) [33,68]. SOD is highly responsive even to slight increases in ROS levels [69] and is rapidly activated under stress, making it an early and sensitive marker of oxidative imbalance [70]. The higher SOD activity observed in Kecskemét CSS-treated plants may be related to the approximately twofold higher nitrogen content in Kecskemét CSS (20.62 g kg^−1^ dry weight) compared to Debrecen CSS, which could have induced different metabolic responses in the plants. This result further highlights the combined influence of sludge origin and applied concentration on the measured parameters. Furthermore, Kecskemét CSS contains higher levels of nickel, which is known to influence urease activity in soil, an important factor for nutrient cycling and agricultural productivity [71]. Higher urease activity typically results in increased nitrogen availability. Additionally, a study in soybeans showed that nickel application enhanced nitrogen accumulation [72]. Initially, the high nitrogen content may have caused stress in the plants, leading to elevated SOD activity and increased chlorophyll-b synthesis. However, over time, the plants may have acclimatized to these conditions. By 35 DAS, the available nickel may have been utilized and nitrogen levels reduced, which could explain the observed decrease in chlorophyll-b content.

The correlation analyses presented in this study revealed distinct physiological and biochemical interactions in maize under composted (CSS) and non-composted sewage sludge (NCSS) treatments. These differences show the contrasting stress responses elicited by the two sludge types and highlight the importance of stabilization through composting in mitigating adverse effects on plant metabolism. In the Debrecen NCSS treatment, plant height was strongly and negatively correlated with several chlorophyll parameters, such as Chl-a (r = −0.961) and total chlorophyll (r = −0.762), while showing a strong positive correlation with APX (r = 0.988) and SOD (r = 0.854), suggesting a stress-induced enhancement of antioxidative enzyme activity (Table S1). Similar trends were found in the Kecskemét NCSS, where height also correlated negatively with chlorophyll parameters such as Chl-a and Chl a/b, and positively with F_v_/F_m_ (r = 0.980) and F_v_/F_o_ (r = 0.979), further emphasizing the stress adaptation response through enhanced PSII efficiency and antioxidative defense (Table S3). Conversely, in CSS treatments (Tables S2 and S4), these stress-related patterns were significantly less pronounced. For example, in Debrecen CSS, Chl-a and Chl-b were positively and significantly correlated with total chlorophyll (r = 0.953 ** and r = 0.965 **, respectively), indicating a more stable pigment profile. The reduced correlation between height and oxidative stress markers (e.g., APX: r = −1.00 in Debrecen CSS (Table S2) vs. r = 0.988 in Debrecen NCSS (Table S1)) supports the idea that composting minimizes oxidative damage, likely due to lower heavy metal bioavailability and a more balanced nutrient release [73,74]. In Kecskemét CSS (Table S4), the Chl-a/Chl-b ratio was significantly negatively correlated with Chl-b (r = −0.898 **) and total chlorophyll (r = −0.212), which might reflect a shift in light-harvesting complex adjustment rather than stress-induced pigment loss. The decreased activity of POD and its inverse significant correlation with Chl-b (r = −0.536 *) suggest that composted sludge reduced oxidative stress, leading to reduced peroxidase-mediated ROS scavenging. Furthermore, photosynthetic parameters like F_v_/F_m_ and F_v_/F_o_ were significantly positively correlated with F_o_ and F_m_ in both CSS treatments, particularly in Debrecen CSS (F_v_/F_m_ and F_m_: r = 0.696 **, F_v_/F_o_ and F_m_: r = 0.690 **) (Table S2), indicating enhanced PSII functionality under CSS. This aligns with findings from Chauhan et al. [55], who summarized that healthier chloroplast structures support higher photosystem efficiency under low-stress conditions. The consistent pattern across both origins suggests that while NCSS treatments may temporarily enhance certain antioxidant activities, they are also associated with reduced chlorophyll levels, disrupted photosynthetic function, and increased stress signaling [75]. Composting not only mitigates these effects but also supports balanced physiological responses, reinforcing previous studies on composted sludge as a safer organic amendment [40,76,77,78].

## 4. Materials and Methods

### 4.1. Experimental Conditions and Treatments

The experimental plants (*Zea mays* L. cv. Armagnac) were grown in a greenhouse under controlled conditions, with temperatures maintained at 25 °C during the day and 20 °C at night, and relative humidity (RH) kept between 65 and 75%. Four sewage sludge treatments were used: Debrecen non-composted sewage sludge, Kecskemét non-composted sewage sludge, Debrecen composted sewage sludge, Kecskemét composted sewages sludge (Debrecen N 47°30′11.2″, E 21°35′49.1″, Kecskemét N 46°53′33.7″, E 19°42′50.1″). Each treatment was applied at four concentrations: 0% test material and 100% soil as a control, 25% test material and 75% soil, 50% test material and 50% soil, and 75% test material and 25% soil (m/m%). For each concentration, seven plants were used in three replicates. Three plants were planted in polyvinyl chloride tubes, with each tube measuring 25 cm in height, 5 cm in diameter, and having a volume of 1570 cm^3^. The plants were watered as needed to avoid water stress. One week after germination, the strongest plant was left in the pot for the duration of the experiment. Since heavy metal is a major problem associated with sewage sludge, the heavy metal composition in these sewage sludges was measured (Table 5).

As a control, the poorly productive Karcagpuszta, Hungary (N 47.25154; E 20.83351) soil was used (Table 6). The first leaf sampling point was performed 21 days after sowing (21 DAS), when the plants were at the 3-leaf stage, and the second was 35 days after sowing (35 DAS), when the plants were at the 5-leaf stage. At each point, the youngest, but fully expanded leaf was sampled. For the destructive measurements, the leaves were harvested and immediately frozen in liquid nitrogen, followed by storage at −80 °C.

### 4.2. Composting Process Explanation

#### 4.2.1. Debrecen

In open-field composting, the maturation period is at least 1.5 months and can be up to 2.5 months during the winter. The same composting area is used for pre-treatment, composting, and post-treatment. During the preparation for composting, foreign materials are removed, lignocellulose is shredded, and any additives are prepared. In the first, aerobic stage of composting, the material placed into windrows is turned every 3–5 days to ensure proper aeration. Once the temperature of the windrows reaches 65 °C and the anaerobic phase begins, it is then sufficient to turn the windrows only once per week. A temperature of 55 °C is maintained for 14 days, during which sterilization processes take place. The materials used for composting include clippings and hay from various herbaceous plants, straw from cereals and legumes, prunings from shrubs and trees, and plant-derived agricultural by-products.

#### 4.2.2. Kecskemét

The composting technology is a closed, aerobic, accelerated maturation process, covered with a semi-permeable GORE™ COVER PLS membrane (W. L. Gore & Associates, Newark, DE, USA). The raw materials used for composting are as follows: digested, dewatered sewage sludge, biowaste (green waste), and fiber residue. The composting process is two-phased, consisting of an intensive 21-day pre-maturation stage followed by a 21-day post-maturation stage. During the intensive phase, hygienization of the compost takes place—as a result of microbial activity, the compost temperature exceeds 60 °C for one week. The use of the semi-permeable membrane allows for an optimal and balanced heat regime within the windrow. This heat retention is complemented by active aeration, ensuring complete and continuous hygienization of the material throughout the process. The combination of the aeration unit and the GORE™ COVER membrane provides uniform heat distribution inside the windrow. Positive pressure is created inside the windrow, which both promotes uniform oxygen distribution and prevents excessive moisture loss. After the 21-day intensive maturation period, the compost is unloaded and transferred to the post-maturation silos. During the post-maturation stage, the transformation processes are completed, and the decomposed organic materials recombine into larger humus molecules.

### 4.3. Determination of Plant Height

The height of the plants was measured from the soil surface to the emergence of the last fully developed leaf using a tape measure in centimeters.

### 4.4. Measurement of the Photosynthetic Pigments

Photosynthetic pigments were quantified destructively using the methods of Moran and Porath [79] and Wellburn [80]. A 50 mg plant sample of leaves was homogenized to a fine paste using pre-chilled mortar and pestle in 5 mL of ice-cold N, N-dimethylformamide. After extraction, the slurry was stored at 4 °C and measured after 72 h on a Nicolet Evolution 300 LC spectrophotometer (Thermo Electric Corporation, Madison, WI, USA) at 480, 647, and 664 nanometer wavelengths. The amounts of chlorophyll-a, chlorophyll-b, and carotenoids were determined using the following formulae:

chlorophyll-a = (11.65 × a664 − 2.69 × b647)

chlorophyll-b = (20.81 × b647 − 4.53 × a664)

carotenoids = (1000 × car480 − 1.28 × a664 − 56.7 × b647)

total chlorophyll = chlorophyll-a + chlorophyll-b

### 4.5. Measurement of Photochemical Efficiency

The in vivo measurement of photosynthetic efficiency was conducted using an OS5p+ pulse-modulated portable chlorophyll fluorometer (Opti-Sciences, Hudson, NY, USA) to assess parameters from the fast phase of chlorophyll fluorescence induction. Before measurements, leaves were dark-adapted for 20 min. During the measurement, the dark-adapted leaves were first illuminated with weak fluorescent light, and the level of the basal fluorescence (F_o_) was measured, then, after the application of a saturating light pulse (8000 μmol m^−2^ s^−1^), the instrument detected the maximum fluorescence (F_m_), which drops back to the F_o_ level after about 20 s in the dark. The difference between Fm and F_o_ is the variable fluorescence (F_v_). The ratio F_v_/F_m_ was used to characterize the maximum photochemical efficiency of PSII, while F_v_/F_o_ is the potential fluorescence [81].

### 4.6. Measurement of the Antioxidant Enzyme Activities

Utilizing the technique outlined by Pukacka and Ratahczak [82], frozen leaf samples (0.2 g) were crushed to a fine paste in 1 mL of 50 mM potassium phosphate buffer (pH 7.0), which also contained 1 mM ethylenediaminetetraacetic acid (EDTA), 0.1% (*v*/*v*) Triton X-100, 1 mM ascorbate, and 2% (*w*/*v*) polyvinylpyrrolidone (PVP). The materials were separated into their parts by centrifuging them at 15,000× *g* for 20 min at 4 °C. The acquired supernatant, which represented the enzyme extract and was transferred to an Eppendorf tube and kept on ice.

Mishra et al. [83] technique was used for the APX assay with modifications. The reaction consisted of 550 µL of 50 mM potassium phosphate buffer (pH 7.0), 200 µL H_2_O_2_ (0.1 mM), 150 µL sodium ascorbate (0.5 mM), 50 µL EDTA (0.1 mM EDTA), and 50 µL sample extract. As a result of ascorbate oxidation, absorbance decreased for five minutes at 20 °C at 290 nm. The blank included extraction buffer instead of the enzyme extract. The extinction coefficient of 2.8 mM^−1^ cm^−1^ was used.

The guaiacol peroxidase activity (POD) measurement was described by Zieslin and Ben-Zaken [84]. To measure tetraguaiacol production, the following reagents were added to a cuvette: 50 µL of 0.2 M H_2_O_2_, 100 µL of 50 mM guaiacol, 340 µL of distilled water, 490 µL of 80 mM phosphate buffer (pH 5.5), and 20 µL of the sample extract. The reaction mixture was incubated at 30 °C, and absorbance was measured at 470 nm over a period of three minutes. For the blank, the sample extract was replaced with 50 mM phosphate buffer. The estimated concentration of tetraguaiacol was determined using an extinction coefficient of 26.6 mM^−1^ cm^−1^.

Superoxide dismutase (SOD) activity was assessed following the protocol described by Giannopolities and Ries [85] and Beyer and Fridovich [86]. One unit of SOD activity was defined as the amount of enzyme required to inhibit 50% of the light-induced reduction of nitroblue tetrazolium (NBT) compared to control tubes without the plant extract. The reaction mixture consisted of 25 µL of the plant extract (supernatant), 25 µL of NBT (9 mM), 25 µL of riboflavin (0.25 mM), 250 µL of methionine (0.16 M), and 2.675 mL of phosphate buffer (50 mM, pH 7.8). This mixture was incubated at room temperature, and absorbance was recorded at 560 nm after 15 min. Blank samples contained 2.7 mL of phosphate buffer without the plant extract, while all other components remained unchanged.

The protein content of the sample extracts was determined using the method described by Bradford [87].

### 4.7. Proline Determination

Proline content was measured according to the method of Carillo and Gibon [88]. Plant samples (0.1 g) were homogenized with 2 mL of 70% (*v*/*v*) ethanol solution and placed on ice. Subsequently, they were centrifuged for 10 min at 15,000× *g* (Heal Force High-Speed Refrigerated Centrifuge, Neofuge 15R China). The supernatant (500 µL) was placed in Eppendorf tubes to which 500 µL of 60% glacial acetic acid, 1% (*w*/*v*) ninhydrin mixture, and 500 µL of 20% (*v*/*v*) ethanol solution were added. The mixtures were then vortexed and heated for 20 min at 95 °C in a Bandelin Sororex Digitec Dt 255 H water bath (Bandelin Electronic GmbH & Co., Berlin, Germany). The samples were then centrifuged at 10,800× *g* for 10 min and placed on ice. The absorbance was then measured at 520 nm using a Nicolet Evolution 300 LC spectrophotometer (Electric Corporation, Madison, WI, USA). Proline content was determined using a proline standard curve.

### 4.8. Statistical Analysis

IBM SPSS Statistics 25 (Armonk, NY, USA) software was used to conduct the statistical analysis. The data normalcy test was conducted using the Kolmogorov–Smirnov and Shapiro–Wilk tests; the findings were examined using a one-way ANOVA, and the Tukey HSD test was used to compare the means. Lowercase letters (a, b, c, and d), * and ** symbols were used in the manuscript to indicate significance. In addition, the Pearson correlation method was used for the correlation analysis.

## 5. Conclusions

In conclusion, the results of this study largely support the hypothesis that increasing concentrations of composted sewage sludge (CSS) have a more favorable effect on maize’s physiological, morphological, and biochemical characteristics compared to non-composted sewage sludge (NCSS). This study demonstrated that both composted (CSS) and non-composted sewage sludge (NCSS) from two distinct origins (Debrecen and Kecskemét) influenced maize growth, pigment composition, oxidative stress responses, and antioxidant enzyme activities in a concentration- and source-dependent manner.

Overall, NCSS from Debrecen significantly improved plant height and stress-related biochemical responses, with the 25% and 50% application rates being the most effective. At 21 and 35 DAS, these treatments enhanced plant height by up to 1.52-fold and increased proline, POD, and APX activities, indicating elevated stress mitigation capacity. However, higher concentrations (75%) often induced excessive oxidative stress, particularly evident in elevated antioxidant enzyme activities and inconsistent growth outcomes. Kecskemét NCSS also stimulated positive responses, but only at lower concentrations—specifically 25%—beyond which (50% and 75%) plant height declined markedly, and photochemical efficiency was negatively impacted. In contrast, Debrecen CSS proved beneficial across all concentrations in promoting shoot height, particularly at 20% and 75%, and enhanced pigment biosynthesis at later growth stages. However, it suppressed antioxidant enzyme activity and caused early reductions in chlorophyll content, suggesting potential initial stress followed by acclimation. Kecskemét CSS was most effective at moderate concentrations (25–50%), improving chlorophyll content and SOD activity without inducing adverse effects on growth. Notably, all CSS treatments from both origins reduced peroxidase activity at 35 DAS, suggesting a systemic shift in oxidative defense strategies.

Based on the combined evaluation of growth, pigment accumulation, photochemical efficiency, and antioxidant responses 25% application rate is recommended for both CSS and NCSS, particularly from Kecskemét, where higher doses impaired plant performance.

For Debrecen sludge, a 25–50% range appears optimal for NCSS, while 20–75% CSS can be applied depending on the desired physiological effect, though caution is advised due to early pigment suppression at lower CSS doses.

These findings highlight the importance of considering sludge origin, composting status, concentration, and application timing when evaluating the physiological effects of organic amendments. The strong interaction effects and correlated responses among key parameters suggest that sludge applications can be optimized not only for nutrient delivery but also to mitigate stress and support plant health. Future studies should explore longer-term physiological outcomes and the potential of integrated pigment-antioxidant markers as indicators for amendment efficacy and safety.

## Figures and Tables

**Table 1:** Effects of non-composted sludge originating from two wastewater treatment plants (Debrecen and Kecskemét) on maize chlorophyll and carotenoid content (mg g^−1^ FW).

Treatments	Chl-a	Chl-b	Car	Chl a/b	Total chl	Total Chl/car
	21 DAYS AFTER SOWING					
Control	12.72 ± 1.74 a	4.76 ± 0.65 a	3.96 ± 0.26 a	2.71 ± 0.44 a	19.12 ± 2.66 a	2.68 ± 0.02 a
Debr 25%	17.93 ± 3.74 a	5.97 ± 2.25 a	8.52 ± 1.49 a	3.21 ± 0.72 a	23.90 ± 5.85 a	2.77 ± 0.22 a
Debr 50%	21.14 ± 1.49 a	9.56 ± 1.90 a	10.35 ± 0.30 a	2.30 ± 0.57 a	30.70 ± 0.57 b	2.97 ± 0.11 a
Debr 75%	17.32 ± 1.59 a	4.53 ± 0.49 a	8.19 ± 0.63 a	3.53 ± 0.55 a	22.38 ± 2.71 a	2.71 ± 0.02 a
Kecs 25%	20.29 ± 0.79 b	20.28 ± 1.09 b	6.09 ± 0.70 c	1.01 ± 0.05 a	39.51 ± 2.71 b	6.53 ± 0.95 b
Kecs 50%	17.59 ± 3.68 b	20.11 ± 0.59 b	6.08 ± 0.07 c	0.85 ± 0.12 a	38.22 ± 5.88 b	6.31 ± 0.95 b
Kecs 75%	20.16 ± 1.59 b	21.21 ± 1.89 b	5.04 ± 0.18 b	0.97 ± 0.02 a	41.37 ± 3.28 b	7.89 ± 0.54 c
	35 DAYS AFTER SOWING					
Control	9.51 ± 1.38 a	3.93 ± 0.33 a	6.61 ± 0.74 a	3.82 ± 0.06 a	13.26 ± 1.65 a	2.57 ± 0.11 a
Debr 25%	21.64 ± 1.10 a	9.08 ± 1.04 a	9.99 ± 0.61 a	2.38 ± 0.07 a	30.72 ± 1.60 b	3.08 ± 0.04 a
Debr 50%	22.64 ± 0.59 a	8.61 ± 1.05 a	10.30 ± 0.31 a	2.46 ± 0.04 a	29.99 ± 2.19 b	2.99 ± 0.02 a
Debr 75%	19.38 ± 2.17 a	8.36 ± 2.41 a	9.18 ± 0.10 a	2.52 ± 0.88 a	27.74 ± 1.64 b	3.00 ± 0.07 a
Kecs 25%	22.46 ± 0.78 c	9.96 ± 1.72 c	12.61 ± 0.56 b	2.09 ± 0.16 a	31.96 ± 2.56 c	2.54 ± 0.19 b
Kecs 50%	19.76 ± 0.94 bc	7.59 ± 0.74 b	12.04 ± 0.96 b	2.52 ± 0.45 ab	26.49 ± 1.78 bc	2.13 ± 0.01 a
Kecs 75%	18.11 ± 3.42 b	5.71 ± 0.94 ab	11.25 ± 1.64 b	2.97 ± 0.13 b	24.85 ± 5.81 b	2.19 ± 0.26 a

Values in columns are means ± standard deviation (*n* = 5). Different lowercase letters show significant differences among treatments (*p* ≤ 0.05), Debr: Debrecen, Kecs: Kecskemét, Chl-a: chlorophyll-a, chl-b: chlorophyll-b, Car: carotenoids, FW: fresh weight.

**Table 2:** Effects of composted sludge originating from two wastewater treatment plants (Debrecen and Kecskemét) on maize chlorophyll and carotenoid content (mg g^−1^ FW).

Treatments	Chl-a	Chl-b	Car	Chl a/b	Total Chl	Total Chl/car
	21 DAYS AFTER SOWING					
Control	12.72 ± 1.74 a	4.76 ± 0.65 b	3.96 ± 0.26 a	2.71 ± 0.44 a	19.12 ± 2.66 a	2.68 ± 0.02 a
Debr 25%	9.93 ± 0.41 a	3.69 ± 0.39 a	4.01 ± 0.38 a	2.71 ± 0.32 a	13.62 ± 0.55 b	3.39 ± 0.07 b
Debr 50%	10.83 ± 1.54 a	3.50 ± 0.20 a	4.46 ± 0.77 a	3.08 ± 0.34 a	14.34 ± 1.69 b	3.24 ± 0.17 b
Debr 75%	11.96 ± 1.95 a	4.13 ± 0.57 ab	4.51 ± 0.92 a	2.89 ± 0.23 a	16.10 ± 2.48 ab	3.60 ± 0.29 b
Kecs 25%	18.99 ± 1.86 b	7.04 ± 0.02 b	9.04 ± 0.45 b	2.80 ± 0.21 a	26.41 ± 1.34 b	2.98 ± 0.14 ab
Kecs 50%	20.81 ± 0.33 b	7.89 ± 1.45 b	8.40 ± 1.11 b	2.64 ± 0.35 a	28.37 ± 2.96 b	3.42 ± 0.58 b
Kecs 75%	19.43 ± 0.43 b	6.18 ± 0.37 ab	8.47 ± 0.99 b	2.99 ± 0.17 a	25.49 ± 1.36 b	2.83 ± 0.07 ab
	35 DAYS AFTER SOWING					
Control	9.51 ± 1.38 a	3.93 ± 0.33 a	6.61 ± 0.74 a	3.82 ± 0.06 b	13.26 ± 1.65 a	2.57 ± 0.11 a
Debr 25%	19.32 ± 2.59 b	6.60 ± 1.09 b	8.56 ± 0.98 b	2.94 ± 0.11 a	25.92 ± 1.77 b	3.02 ± 0.09 b
Debr 50%	18.43 ± 1.71 b	5.95 ± 0.25 b	8.17 ± 0.57 ab	3.04 ± 0.31 a	24.50 ± 1.77 b	2.99 ± 0.08 b
Debr 75%	18.12 ± 2.72 b	5.94 ± 0.24 b	7.95 ± 1.07 ab	2.85 ± 0.39 a	24.52 ± 3.51 b	3.08 ± 0.04 b
Kecs 25%	19.39 ± 1.78 b	9.13 ± 0.37 b	9.47 ± 0.73 b	2.16 ± 0.10 a	28.79 ± 3.31 b	3.03 ± 0.13 b
Kecs 50%	18.52 ± 1.26 b	7.48 ± 1.14 b	8.72 ± 0.59 b	2.52 ± 0.43 ab	26.00 ± 1.58 b	2.98 ± 0.07 b
Kecs 75%	20.09 ± 1.36 b	8.30 ± 1.51 b	9.05 ± 1.03 b	2.47 ± 0.39 ab	28.39 ± 2.39 b	3.06 ± 0.04 b

Values in columns are means ± standard deviation (*n* = 5). Different lowercase letters show significant differences among treatments (*p* ≤ 0.05), Debr: Debrecen, Kecs: Kecskemét, Chl-a: chlorophyll-a, chl-b: chlorophyll-b, Car: carotenoids, FW: fresh weight.

**Table 3:** The photosynthesis efficiency parameters of maize treated with non-composted sludge originating from two wastewater treatment plants (Debrecen and Kecskemét), 21 and 35 days after sowing.

Treatments	Fo	Fm	Fv	Fv/Fm	Fv/Fo
	21 DAYS AFTER SOWING				
Control	155.00 ± 8.33 a	767.50 ± 47.09 a	612.50 ± 38.87 b	0.78 ± 0.00 a	3.59 ± 0.05 a
Debr 25%	145.75 ± 2.63 a	666.40 ± 13.14 a	523.60 ± 10.45 a	0.78 ± 0.01 a	3.67 ± 0.19 a
Debr 50%	143.67 ± 3.21 a	689.60 ± 56.67 a	545.00 ± 44.39 a	0.78 ± 0.01 a	3.77 ± 0.17 a
Debr 75%	142.50 ± 3.11 a	691.00 ± 6.25 a	548.00 ± 8.71 a	0.79 ± 0.01 a	3.89 ± 0.16 a
Kecs 25%	160.00 ± 5.32 a	727.00 ± 38.66 a	558.25 ± 14.66 ab	0.78 ± 0.01 c	3.67 ± 0.18 c
Kecs 50%	164.80 ± 3.82 ab	705.20 ± 39.97 a	540.40 ± 37.15 a	0.76 ± 0.00 b	3.28 ± 0.18 ab
Kecs 75%	186.40 ± 19.11 b	752.00 ± 24.07 a	570.50 ± 12.79 ab	0.75 ± 0.01 a	3.14 ± 0.25 b
	35 DAYS AFTER SOWING				
Control	165.00 ± 9.98 a	815.80 ± 23.38 a	650.80 ± 13.44 a	0.79 ± 0.01 a	3.95 ± 0.16 a
Debr 25%	161.67 ± 2.52 a	802.00 ± 43.83 a	641.20 ± 33.06 a	0.80 ± 0.00 a	3.99 ± 0.10 a
Debr 50%	161.25 ± 1.70 a	801.40 ± 29.45 a	641.60 ± 27.01 a	0.80 ± 0.01 a	4.01 ± 0.13 a
Debr 75%	157.60 ± 7.37 a	776.60 ± 39.70 a	616.33 ± 8.62 a	0.80 ± 0.01 a	3.93 ± 0.15 a
Kecs 25%	217.80 ± 7.89 b	787.80 ± 170.83 a	570.00 ± 170.93 a	0.71 ± 0.01 a	2.62 ± 0.80 a
Kecs 50%	218.20 ± 27.49 b	735.00 ± 10.15 a	507.40 ± 68.67 a	0.70 ± 0.05 a	2.38 ± 0.57 a
Kecs 75%	240.80 ± 12.78 b	782.00 ± 36.62 a	541.20 ± 36.54 a	0.69 ± 0.02 a	2.25 ± 0.21 a

Values in columns are means ± standard deviation (*n* = 5). Different lowercase letters show significant differences among treatments (*p* ≤ 0.05), Debr: Debrecen, Kecs: Kecskemét, F_o_: basal fluorescence, F_m_: maximal fluorescence, F_v_: variable fluorescence.

**Table 4:** The photosynthesis efficiency parameters of maize treated with composted sludge originating from two wastewater treatment plants (Debrecen and Kecskemét), 21 and 35 days after sowing.

Treatments	Fo	Fm	Fv	Fv/Fm	Fv/Fo
	21 DAYS AFTER SOWING				
Control	155.00 ± 8.33 b	767.50 ± 47.09 b	612.50 ± 38.87 b	0.78 ± 0.00 a	3.59 ± 0.05 a
Debr 25%	145.80 ± 5.93 b	734.40 ± 29.65 ab	588.60 ± 24.82 ab	0.80 ± 0.00 b	4.02 ± 0.03 b
Debr 50%	153.50 ± 4.12 b	733.80 ± 33.30 ab	583.20 ± 26.28 ab	0.80 ± 0.00 b	3.87 ± 0.06 b
Debr 75%	132.00 ± 7.21 a	669.80 ± 44.76 ab	537.80 ± 39.32 a	0.80 ± 0.00 b	3.98 ± 0.07 b
Kecs 25%	149.80 ± 13.61 ab	739.75 ± 18.45 a	595.00 ± 11.28 a	0.80 ± 0.01 b	4.09 ± 0.18 b
Kecs 50%	156.25 ± 3.30 ab	825.20 ± 53.69 b	663.40 ± 42.25 b	0.80 ± 0.01 b	4.11 ± 0.15 b
Kecs 75%	161.75 ± 4.03 b	803.60 ± 39.66 ab	645.00 ± 32.02 ab	0.80 ± 0.00 b	4.07 ± 0.05 b
	35 DAYS AFTER SOWING				
Control	165.00 ± 9.98 a	815.80 ± 23.38 a	650.80 ± 13.44 a	0.79 ± 0.01 a	3.95 ± 0.16 a
Debr 25%	167.80 ± 7.19 a	875.40 ± 42.40 ab	707.60 ± 35.45 ab	0.81 ± 0.00 ab	4.22 ± 0.01 ab
Debr 50%	190.75 ± 3.50 b	937.33 ± 17.24 b	756.00 ± 10.15 b	0.81 ± 0.01 ab	4.25 ± 0.26 ab
Debr 75%	164.80 ± 11.79 a	897.20 ± 65.23 ab	732.40 ± 53.48 ab	0.82 ± 0.00 b	4.44 ± 0.03 b
Kecs 25%	159.25 ± 10.21 a	840.00 ± 69.66 a	680.75 ± 60.19 a	0.81 ± 0.01 b	4.27 ± 0.17 b
Kecs 50%	154.20 ± 5.89 a	809.40 ± 22.18 a	655.20 ± 17.67 a	0.81 ± 0.00 b	4.26 ± 0.02 ab
Kecs 75%	149.00 ± 12.62 a	782.50 ± 87.41 a	633.50 ± 74.88 a	0.81 ± 0.01 ab	4.24 ± 0.15 ab

Values in columns are means ± standard deviation (*n* = 5). Different lowercase letters show significant differences among treatments (*p* ≤ 0.05), Debr: Debrecen, Kecs: Kecskemét, F_o_: basal fluorescence, F_m_: maximal fluorescence, F_v_: variable fluorescence.

**Table 5:** The heavy metal content of non-composted sewage sludge and composted sewage sludge from two different sources.

	Element Content (mg kg^−1^ Dry Material)					
	Debrecen		Kecskemét		Element Content Concerning the Permitted Limit	
Elements	NCSS	CSS	NCSS	CSS	CSS	NCSS
Cadmium	1.00	<1	<1	<1	5	10
Cobalt	4.39	4.72	6.06	4.64	50	50
Chromium	51.3	51.1	71.1	48.1	350	1000
Copper	218	139	199	119	750	1000
Mercury	0.76	0.91	0.25	0.28	5	10
Nickel	22.4	19.5	49	28	100	200
Lead	26.6	24	14.7	28.2	400	750

NCSS: non-composted sewage sludge, CSS: composted sewage sludge.

**Table 6:** The main parameters of the soil used.

Tests Performed	Soil Test Results	Extended Measurement Uncertainty
pH value (KCl)	6.3	±5%
Gold-bonded number	50	±10%
Total water soluble salinity [m/m%]	0.06	±10%
Hydrochloric acid lime [m/m%]	0.21	±10%
Humus content [m/m%]	2.9	±10%
(nitrate + nitrite)-N (KCl soluble) [mg/kg]	4.2	±5%
Phosphorus pentoxide (AL soluble) [mg/kg]	211	±10%
Potassium oxide (AL soluble) [mg/kg]	419	±10%
Sodium (AL soluble) [mg/kg]	445	±10%
Magnesium (KCl-soluble) [mg/kg]	647	±10%
Sulfated sulfur (KCl-soluble) [mg/kg]	7.9	±5%
Cadmium (EDTA-Na_2_ soluble) [mg/kg]	<1	±10%
Cobalt (EDTA-Na_2_ soluble) [mg/kg]	<1	±10%
Chromium (EDTA-Na_2_ soluble) [mg/kg]	<1	±10%
Mercury (EDTA-Na_2_ soluble) [mg/kg]	<1	±10%
Nickel (EDTA-Na_2_ soluble) [mg/kg]	<1	±10%
Lead (EDTA-Na_2_ soluble) [mg/kg]	<1	±10%
Zinc (EDTA-Na_2_ soluble) [mg/kg]	0.9	±10%
Copper (EDTA-Na_2_ soluble) [mg/kg]	9	±10%
Manganese (EDTA-Na_2_ soluble) [mg/kg]	201	±10%

## Data Availability

Data are contained within the article or Supplementary Materials.

## Supplementary Materials

The following supporting information can be downloaded at: https://www.mdpi.com/article/10.3390/plants14131955/s1, Table S1: Correlation analysis for Debrecen NCSS combined the three concentrations (25%, 50%, and 75% m/m%), Table S2: Correlation analysis for Debrecen CSS combined the three concentrations (25%, 50%, and 75% m/m%), Table S3: Correlation analysis for Kecskemét NCSS combined the three concentrations (25%, 50%, and 75% m/m%), Table S4: Correlation analysis for Kecskemét CSS combined the three concentrations (25%, 50%, and 75% m/m%).
