# Supplementary material for: Comparative Effects of Non-Composted and Composted Sewage Sludge from Wastewater Treatment Plants on the Physiological and Antioxidative Responses of Maize
Source: Plants (Basel). 2025 Jun 26;14(13):1955. doi: 10.3390/plants14131955 (PMC12251873; doi:10.3390/plants14131955)
Supplement: Supplementary file 1 [file plants-14-01955-s001.zip › plants-3646462-supplementary.pdf]

Table S1: Correlation analysis for Debrecen NCSS combined the three concentrations (25%, 50%, and 75% m/m%)
*indicates significance at $p < 0.05$; ** indicates significance at $p < 0.01$

Char 1	Char 2															
	Height	Chl-a	Chl-b	Total chl	Chl a/b	Car	Total chl/car	Fo	Fm	Fv	Fv/Fm	Fv/Fo	Prolin	APX	POD	SOD
**Height**	1	-0.961	-0.599	-0.762	0.485	-0.818	-0.703	-0.388	-1.0	-1.0	1.0	1.0	-0.375	0.988	-0.716	0.854
**Chl-a**	-0.961	1	0.536**	0.852**	-0.423*	0.938**	0.634**	0.528*	0.392*	0.388	0.125	0.126	-0.064	-0.017	-0.098	0.247
**Chl-b**	-0.599	0.536**	1	0.663**	-0.973**	0.762**	0.877**	0.386	0.327	0.360	0.032	0.042	0.259	-0.226	0.003	0.175
**Total Chl**	-0.762	0.852**	0.663**	1	-0.511**	0.937**	0.597**	0.267	0.163	0.148	-0.138	-0.143	-0.045	-0.174	-0.089	0.135
**Chl a/b**	0.485	-0.423*	-0.973**	-0.511**	1	-0.657**	-0.865**	-0.341	-0.321	-0.369	0.005	-0.005	-0.252	0.213	-0.073	-0.219
**Car**	-0.818	0.938**	0.762**	0.937**	-0.657**	1	0.734**	0.541	0.351	0.367	0.065	0.072	0.150	-0.059	-0.102	0.204
**Total chl/car**	-0.703	0.634**	0.877**	0.597**	-0.865**	0.734**	1	0.513*	0.533*	0.539**	0.171	0.175	0.265	-0.042	0.127	0.208
**Fo**	-0.388	0.528*	0.386	0.267	-0.341	0.541*	0.513*	1	0.919**	0.885**	0.513*	0.513*	0.150	0.131	0.188	0.410
**Fm**	-1.0	0.392*	0.327	0.163	-0.321	0.351	0.533**	0.919**	1	0.996**	0.586**	0.585**	0.068	-0.012	0.366	0.096
**Fv**	-1.0	0.388	0.360	0.148	-0.369	0.367	0.539**	0.885**	0.996**	1	0.626**	0.624**	0.055	-0.029	0.319	0.104
**Fv/Fm**	1.0	0.125	0.032	-0.138	0.005	0.065	0.171	0.513*	0.586**	0.626**	1	0.999**	-0.313	0.357	0.424*	-0.091
**Fv/Fo**	1.0	0.126	0.042	-0.143	-0.005	0.072	0.175	0.513*	0.585**	0.624**	0.999**	1	-0.301	0.228	0.420*	-0.010
**Prolin**	-0.375	-0.064	0.259	-0.045	-0.252	0.150	0.265	0.150	0.068	0.055	-0.313	-0.301	1	0.102	0.114	0.023
**APX**	0.988	-0.017	-0.226	-0.174	0.213	-0.059	-0.042	0.131	-0.012	-0.029	0.357	0.355	0.102	1	0.597**	-0.377
**POD**	-0.716	-0.098	0.003	-0.089	-0.073	-0.102	0.127	0.188	0.366	0.319	0.424*	0.420*	0.114	0.597**	1	-0.441*
**SOD**	0.854	0.247	0.175	0.135	-0.219	0.204	0.208	0.410	0.096	0.104	-0.009	-0.010	0.023	-0.377	0.114	1

Table S2: Correlation analysis for Debrecen CSS combined the three concentrations (25%, 50%, and 75% m/m%)
*indicates significance at $p < 0.05$; ** indicates significance at $p < 0.01$

Char 1	Char 2															
	**Height**	**Chl-a**	**Chl-b**	**Total chl**	**Chl a/b**	**Car**	**Total chl/car**	**Fo**	**Fm**	**Fv**	**Fv/Fm**	**Fv/Fo**	**Prolin**	**APX**	**POD**	**SOD**
**Height**	1	-0.858	-1.0	-0.619	0.176	-0.888	1.0	-0.911	-0.684	-0.602	0.761	0.749	1.0	-1.00	1.00	-0.213
**Chl-a**	-0.858	1	0.939**	0.953**	0.253	0.987**	-0.651**	0.221	0.355	0.351	0.136	0.095	0.404*	0.100	0.036	0.527**
**Chl-b**	-1.0	0.939**	1	0.965**	0.021	0.946**	-0.604**	0.139	0.319	0.319	0.171	0.129	0.530**	0.240	0.086	0.475*
**Total Chl**	-0.619	0.953**	0.965**	1	0.258	0.943**	-0.640**	0.207	0.342	0.338	0.142	0.098	0.492**	0.175	0.104	0.515*
**Chl a/b**	0.176	0.253	0.021	0.258	1	0.204	-0.248	0.275	0.138	0.122	-0.164	-0.200	0.017	-0.098	0.199	0.223
**Car**	-0.888	0.987**	0.946**	0.943**	0.204	1	-0.744**	0.269	0.401*	0.395*	0.151	0.113	0.401*	0.099	0.028	0.488*
**Total chl/car**	1.0	-0.651**	-0.604**	-0.640**	-0.248	-0.744**	1	-0.520**	-0.532**	-0.512**	-0.107	-0.069	-0.281	0.043	-0.074	-0.231
**Fo**	-0.911	0.221	0.139	0.207	0.275	0.269	-0.520**	1	0.951**	0.930**	0.482*	0.476*	0.206	0.081	0.397	0.181
**Fm**	-0.684	0.355	0.319	0.342	0.138	0.401*	-0.532**	0.951**	1	0.998**	0.696**	0.690**	0.361	0.100	0.480*	0.230
**Fv**	-0.602	0.351	0.319	0.338	0.122	0.395*	-0.512**	0.930**	0.998**	1	0.734**	0.728**	0.381	0.113	0.483*	0.244
**Fv/Fm**	0.761	0.136	0.171	0.142	-0.164	0.151	-0.107	0.482*	0.696**	0.734**	1	0.999*	0.573**	0.278	0.527*	0.340
**Fv/Fo**	0.749	0.095	0.129	0.098	-0.200	0.113	-0.069	0.476*	0.690**	0.728**	0.999**	1	0.570	0.282	0.489*	0.276
**Prolin**	1.0	0.404*	0.530**	0.492**	0.017	0.401*	-0.281	0.206	0.361	0.381	0.573**	0.570**	1	0.494**	0.466*	0.288
**APX**	-1.0	0.100	0.240	0.175	-0.098	0.099	0.043	0.081	0.100	0.113	0.278	0.282	0.494**	1	0.225	0.253
**POD**	1.0	0.036	0.086	0.104	0.199	0.028	-0.074	0.397	0.480*	0.493*	0.528*	0.489*	0.466*	0.225	1	0.482*
**SOD**	-0.213	0.527**	0.475*	0.515*	0.223	0.488*	-0.231	0.181	0.230	0.244	0.340	0.276	0.288	0.253	0.482*	1

Table S3: Correlation analysis for Kecskemét NCSS combined the three concentrations (25%, 50%, and 75% m/m%)
*indicates significance at $p < 0.05$; ** indicates significance at $p < 0.01$

Char 1	Char 2															
	**Height**	**Chl-a**	**Chl-b**	**Total chl**	**Chl a/b**	**Car**	**Total chl/car**	**Fo**	**Fm**	**Fv**	**Fv/Fm**	**Fv/Fo**	**Prolin**	**APX**	**POD**	**SOD**
**Height**	1	-0.083	0.213	0.203	-0.223	-0.187	0.145	-0.661	0.447	-1.0	0.980	0.979	0.524	-0.978	-0.997*	-0.743
**Chl-a**	-0.083	1	0.177	0.440*	-0.060	0.216	0.028	-0.102	0.032	0.035	0.054	0.121	0.194	0.076	-0.101	-0.314
**Chl-b**	0.213	0.177	1	0.751**	-0.967**	-0.886**	0.957**	-0.301	-0.170	-0.018	0.302	0.334	0.125	-0.188	-0.197	-0.170
**Total Chl**	0.203	0.440*	0.751**	1	-0.509**	-0.548**	0.618**	-0.262	0.130	0.250	0.323	0.319	0.007	-0.073	-0.046	-0.286
**Chl a/b**	-0.223	-0.060	-0.967**	-0.509**	1	0.839**	-0.899**	0.248	0.054	0.005	-0.171	-0.210	-0.371	0.087	0.077	0.248
**Car**	-0.187	0.216	-0.886**	-0.548**	0.839**	1	-0.943**	0.197	0.020	-0.017	-0.256	-0.288	0.059	-0.021	0.218	0.169
**Total chl/car**	0.145	0.028	0.957**	0.618**	-0.899**	-0.943**	1	-0.277	-0.093	-0.042	0.252	0.281	0.033	0.048	-0.197	-0.281
**Fo**	-0.661	-0.102	-0.301	-0.262	0.248	0.197	-0.277	1	0.311	-0.148	-0.802**	-0.823**	0.048	0.396*	0.734**	0.082
**Fm**	0.447	0.032	-0.170	0.130	0.054	0.020	-0.093	0.311	1	0.896**	0.243	0.204	-0.053	0.279	0.401*	0.019
**Fv**	-1.0	0.035	-0.018	0.250	0.005	-0.017	-0.042	-0.148	0.896**	1	0.681**	0.644**	-0.071	-0.016	-0.004	0.092
**Fv/Fm**	0.980	0.054	0.302	0.323	-0.171	-0.256	0.252	-0.802**	0.243	0.681**	1	0.990**	-0.126	-0.318	-0.574**	0.077
**Fv/Fo**	0.979	0.121	0.334	0.319	-0.210	-0.288	0.281	-0.823**	0.204	0.644**	0.990**	1	-0.052	-0.349	-0.584**	0.043
**Prolin**	0.524	0.194	0.125	0.007	-0.371	0.059	0.033	0.048	-0.053	-0.071	-0.126	-0.052	1	-0.268	0.025	-0.073
**APX**	-0.978	0.076	-0.188	-0.073	0.087	-0.021	0.048	0.396*	0.279	-0.016	-0.318	-0.349	-0.268	1	0.275	-0.290
**POD**	-0.997*	-0.101	-0.197	-0.046	0.077	0.218	-0.197	0.734**	0.401*	-0.004	-0.574**	-0.584**	0.025	0.275	1	0.201
**SOD**	-0.743	-0.314	-0.170	-0.286	0.248	0.169	-0.281	0.082	0.019	0.092	0.077	0.043	-0.073	-0.290	0.201	1

Table S4: Correlation analysis for Kecskemét CSS combined the three concentrations (25%, 50%, and 75% m/m%)
*indicates significance at $p < 0.05$; ** indicates significance at $p < 0.01$

Char 1	Char 2															
	**Height**	**Chl-a**	**Chl-b**	**Total chl**	**Chl a/b**	**Car**	**Total chl/car**	**Fo**	**Fm**	**Fv**	**Fv/Fm**	**Fv/Fo**	**Prolin**	**APX**	**POD**	**SOD**
**Height**	1	0.175	1.0	0.878	-1.0	0.210	-1.0	0.614	0.436	0.331	-0.774	-0.779	0.812	-0.066	-0.072	0.415
**Chl-a**	0.175	1	0.228	0.556**	0.247	0.556**	0.398	-0.092	0.026	0.001	-0.100	-0.109	-0.189	-0.476*	-0.215	0.266
**Chl-b**	1.0	0.228	1	0.324	-0.898**	0.466*	0.297	0.103	-0.002	-0.033	-0.263	-0.263	0.091	0.211	-0.536*	-0.019
**Total Chl**	0.878	0.556**	0.324	1	-0.212	0.385*	-0.068	-0.045	-0.008	-0.032	-0.170	-0.172	0.186	0.034	-0.372	0.148
**Chl a/b**	-1.0	0.247	-0.898**	-0.212	1	-0.340	-0.075	-0.044	0.135	0.165	0.347	0.347	-0.171	-0.306	0.436*	0.069
**Car**	0.210	0.556**	0.466*	0.385*	-0.340	1	-0.463*	-0.049	-0.083	-0.088	-0.011	-0.010	0.156	-0.082	-0.133	-0.130
**Total chl/car**	-1.0	0.398	0.297	-0.068	-0.075	-0.463*	1	0.053	0.197	0.193	0.057	0.060	-0.067	0.165	-0.306	0.358
**Fo**	0.614	-0.092	0.103	-0.045	-0.044	-0.049	0.053	1	0.900**	0.859**	-0.030	-0.009	-0.349	0.149	-0.078	0.107
**Fm**	0.436	0.026	-0.002	-0.008	0.135	-0.083	0.197	0.900**	1	0.995**	0.289	0.314	-0.301	0.149	0.136	0.121
**Fv**	0.331	0.001	-0.033	-0.033	0.165	-0.088	0.193	0.859**	0.995**	1	0.375	0.399*	-0.301	0.175	0.159	0.104
**Fv/Fm**	-0.774	-0.100	-0.263	-0.170	0.347	-0.011	0.057	-0.030	0.289	0.375	1	0.999**	-0.036	0.208	0.306	-0.102
**Fv/Fo**	-0.779	-0.109	-0.263	-0.172	0.347	-0.010	0.060	-0.009	0.314	0.399*	0.999**	1	-0.047	0.206	0.301	-0.102
**Prolin**	0.812	-0.189	0.091	0.186	-0.171	0.156	-0.067	-0.349	-0.301	-0.301	-0.036	-0.047	1	0.159	0.045	0.180
**APX**	-0.066	-0.476*	0.211	0.034	-0.306	-0.082	0.165	0.149	0.149	0.175	0.208	0.206	0.159	1	0.177	0.225
**POD**	-0.072	-0.215	-0.536*	-0.372	-0.436*	-0.133	-0.306	-0.078	0.136	0.159	0.306	0.301	0.045	0.177	1	-0.017
**SOD**	0.415	0.266	-0.019	0.148	-0.069	-0.130	0.358	0.107	0.121	0.104	-0.102	-0.102	0.180	0.225	-0.017	1
